# Periodontitis and systemic diseases: insights into the correlation, mechanisms, and clinical implications

**DOI:** 10.3389/fimmu.2026.1777955

**Published:** 2026-03-11

**Authors:** Zhina Wu, Yaoyao Zhang, Lishan Wang, Yating Yi, Bingfeng Dai, Huanyun Chen, Fanghong Yang

**Affiliations:** 1 Department of Orthodontics, Weifang People’s Hospital, Shandong Second Medical University, Weifang, Shandong, China; 2 Department of Oral and Maxillofacial Surgery, Weifang People’s Hospital, Shandong Second Medical University, Weifang, Shandong, China; 3 School of Stomatology, Shandong Second Medical University, Weifang, Shandong, China; 4 Weifang Hospital of Traditional Chinese Medicine, Shandong Second Medical University, Weifang, Shandong, China

**Keywords:** clinical implications, correlation, mechanisms, periodontitis, systemic diseases

## Abstract

Periodontitis is a chronic oral infectious inflammatory disease caused by dental plaque, affecting approximately 35% - 50% of adults globally. Far from a localized oral condition, it exerts systemic pathogenic effects through multiple biological conduits. This review synthesizes current evidence on the bidirectional associations between periodontitis and a broad spectrum of systemic disorders, including cardiovascular disease (CVD), diabetes mellitus (DM), respiratory diseases, preterm birth, Alzheimer’s disease (AD), chronic kidney disease (CKD), rheumatoid arthritis (RA), and *Helicobacter pylori* (*H. pylori*) infection. Furthermore, the review delves into the potential pathophysiological mechanisms underpinning these associations, with emphasis on bacterial translocation, systemic inflammation, immune dysregulation, and oxidative stress pathways. The concluding remarks underscore the critical importance of preserving optimal periodontal health as a cornerstone of systemic wellbeing.

## Graphical Abstract

**Figure:**
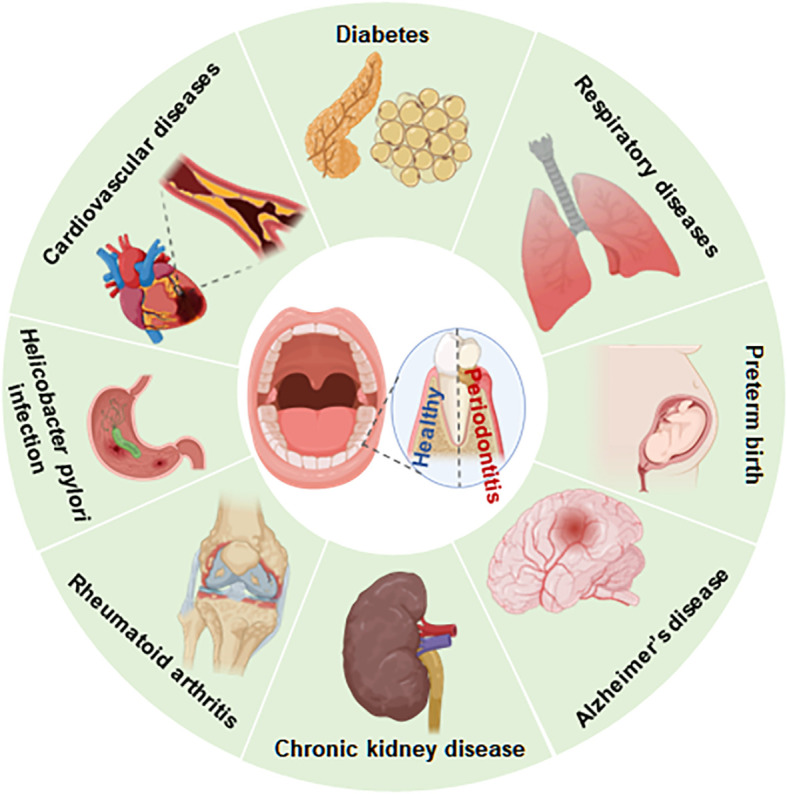

## 1 Introduction

Periodontitis is a plaque-induced inflammatory destruction of tooth-supporting apparatus, ultimately resulting in the loss of periodontal attachment. The 2018 consensus report on the new classification of periodontal diseases stratifies periodontitis into four stages and three grades (**Table 1**). The staging (Stages I–IV) assesses the severity, extent, and complexity of treatment, while grading estimates the risk of progression, prognosis, and potential impacts on systemic health (1). Dental plaque biofilm serves as t the primary etiological agent in the initiation of periodontitis, acting synergistically with environmental, lifestyle, and genetic risk factors to drive disease pathogenesis and progression. The formation of this biofilm involves acquired pellicle development, bacterial adhesion, biofilm maturation, microbial co-aggregation, and dispersion (2, 3). Host immune responses to the biofilm trigger a cascade of inflammatory events that accelerates osteoclastogenesis and alveolar bone resorption, culminating in clinical attachment loss (4, 5). Epidemiological evidence from 2011 to 2020 reveals that periodontitis affects 62% of the adult population at its peak prevalence, with 23.6% of adults experiencing severe forms, making it a principal etiological factor of tooth loss in adults and posing significant challenges to global public health (6).

**Table 1:** Classification of periodontitis.

Stage Grade	Grade A	Grade B	Grade C
Stage I: Initial Severity: Initial; Complexity: Low	Progress slowly, low risk	Progress moderately, moderate risk	Progress quickly, high risk
Stage II: Moderate Severity: Moderate; Complexity: Moderate	Progress slowly, low risk	Progress moderately, moderate risk	Progress quickly, high risk
Stage III: Severe Severity: Severe; Complexity: High Potential for additional tooth loss	Progress slowly, low risk	Progress moderately, moderate risk	Progress quickly, high risk
Stage IV: Advanced Severity: Advanced; Complexity: Very High – Extensive tooth loss, potential edentulism	Progress slowly, low risk	Progress moderately, moderate risk	Progress quickly, high risk

Traditionally, periodontitis has been conceptualized as a localized infectious disorder confined to the oral cavity, primarily affecting the gums, alveolar bone, and supporting periodontal tissues (7). Emerging evidence, however, positions periodontitis as a chronic inflammatory nidus, capable of systemically disseminating pathological effects through hematogenous dissemination and immune-mediated pathways, exhibiting significant comorbidity with various systemic diseases (7, 8). This comprehensive review systematically synthesizes current knowledge on the epidemiological correlations, mechanistic pathways, and graded evidence levels connecting periodontitis with diverse systemic conditions, thereby establishing a scientific foundation for interdisciplinary clinical collaboration. Beyond enumerating disease-specific associations, we also propose an integrative conceptual model that redefines periodontitis as a persistent inflammatory hub driving systemic pathology through both local inflammatory persistence and systemic dissemination.

## 2 Periodontitis and cardiovascular diseases

Cardiovascular disease (CVD) encompasses a broad spectrum of pathophysiological disorders affecting cardiac and vascular function, encompassing atherosclerosis (AS), coronary artery disease, and cerebrovascular accidents. As the leading global cause of mortality, CVD accounts for approximately 30% of all deaths worldwide (9). Periodontitis, a highly prevalent chronic inflammatory condition affecting over 10% of the global population, demonstrates a well-established epidemiological association with CVD (10, 11). Pathophysiologically, periodontitis contributes to endothelial dysfunction and increases susceptibility to primary coronary events. The microbial etiology of periodontitis serves as a potential source for infective endocarditis and valvular pathology (8, 12). Notably, shared genetic predisposition constitutes an additional mechanistic link, with genome-wide association studies identifying susceptibility loci - including CDKN2B-AS1 (ANRIL), PLG, CAMTA1/VAMP3, and VAMP8 - that regulate aberrant inflammatory responses, thereby partially explaining the observed epidemiological correlation between these two conditions (13).

### 2.1 Atherosclerosis

Atherosclerosis (AS) is a chronic inflammatory disease caused by lipid metabolism imbalance, characterized by intimal thickening and plaque formation (14). Compelling evidence substantiates the link between periodontitis and atherosclerotic disease. Periodontitis facilitates the entry of bacteria or their products into the circulation, where they activate multifaceted host inflammatory responses that accelerate the initiation, maturation, and progression of atherosclerotic lesions (15, 16). Notably, oral pathogens including *Aggregatibacter actinomycetemcomitans* (*A. actinomycetemcomitans*), *Prevotella intermedia* (*P. intermedia*) *Fusobacterium nucleatum* (*F. nucleatum*), *Porphyromonas gingivalis* (*P. gingivalis*), and *Treponema denticola* (*T. denticola*), have been identified within atherosclerotic plaques, indicating direct involvement in vascular wall inflammation (17, 18). Animal studies reveal that infection with periodontal pathogens accelerates atherosclerotic formation and lipid deposition. Angioplasty-induced experimental periodontitis, even in the absence of exogenous infection, promotes lipid oxidation in the aorta. Additionally, local oral application of the anti-inflammatory mediator Resolvin E1 significantly reduces arterial plaque formation and periodontal tissue destruction (15, 19). A cross-sectional study involving community residents over 55 years old found that regular dental visits can reduce the risk of periodontitis and tooth loss, thereby helping to control the progression of AS (20). A longitudinal cohort study in Nagasaki Island established a bidirectional association between AS and periodontitis progression. Baseline vascular lesions increased the risk of periodontal progression, while worsening periodontitis also accelerated the development of AS indicators such as intima-media thickness (IMT), both influenced by shared inflammatory mechanisms (21). Systematic reviews and meta-analysis have confirmed a significant link between periodontitis and carotid AS, manifesting as increased IMT and plaque, with reported odds ratio (OR) ranging from approximately 1.6 to 2.0. This finding has clinical significance and supports the presence of a potential pathological connection, underscoring the importance of integrated management of oral and vascular health in clinical practice (22).

However, mendelian randomization (MR) analysis has shown that genetic susceptibility to periodontitis has no significant causal effect on the risk of coronary AS, suggesting that the two conditions may coexist due to shared risk factors such as smoking, or incidental associations. This finding refutes the presence of a direct causal relationship between them (23, 24). Although a definitive causal relationship between periodontitis and AS has not yet been established, existing evidence suggests that controlling periodontal infection may indirectly slow the progression of AS by reducing systemic inflammatory burden. In clinical practice, for high-risk populations for AS including those with coronary artery disease or stroke, standardized periodontal treatments such as scaling, root planning and subgingival debridement may not only enhance oral health but also constitute a key component of a comprehensive strategy for CVD prevention.

### 2.2 Coronary heart disease

Coronary heart disease (CHD) is a leading cause of morbidity and mortality worldwide, attributable to coronary AS, involves narrowing or occlusion of the coronary vessels (25). Multiple studies have identified periodontitis as a significant risk factor for CHD (26, 27). Specifically, moderate to severe periodontitis is strongly associated with both prevalent and incident CHD, suggesting that periodontal disease may act not merely as a correlative factor but also as a potential independent risk factor for CHD (28). In CHD patients, elevated serum antibody levels against periodontal pathogens such as *P. gingivalis* and *A. actinomycetemcomitans* are significantly associated with increased risk of CHD (29, 30). Furthermore, the overall prevalence of periodontitis among CHD patients is significantly higher than that in the general population, with cases predominantly characterized by the moderate-to-severe form (31). These observations are primarily derived from epidemiological data, which fail to establish a direct causal relationship between periodontitis and CHD.

### 2.3 Myocardial infarction

Myocardial infarction (MI), a severe cardiac event caused by abrupt interruption of coronary blood flow, resulting in ischemic necrosis of cardiac muscle cells (12).The development of CHD is associated with multiple risk factors. Evidence indicates that periodontal disease also contributes to MI risk (32), with moderate to severe periodontitis significantly correlating with acute MI (AMI) risk, escalating with severity, implicating its inflammatory burden as a key AMI risk factor (33). Notably, additional studies reveal that severe periodontitis is significantly associated with MI risk in females, whereas the association is weaker in males, indicating potential sex-specific differences (34). Furthermore, patients with severe periodontitis exhibit elevated levels of *P. gingivalis*-specific antibodies, which are significantly associated with certain autoantibodies and MI risk (35). In post-MI patients, periodontitis is also significantly correlated with elevated systemic inflammatory markers, such as C-reactive protein (CRP), suggesting that local periodontal inflammation may exacerbate systemic inflammatory burden and adversely affect cardiovascular prognosis. This underscores the importance of integrated oral-systemic inflammatory management after MI (36).

### 2.4 Stroke

Stroke, a life-threatening cerebrovascular disease characterized by sudden interruption of cerebral perfusion leading to rapid neuronal death, shares overlapping risk factors with periodontitis, including AS, hypertension, and DM (37). Epidemiological studies reveal that individuals with severe and moderate periodontitis exhibit a 2.55-fold and 1.71-fold elevated risk of stroke, respectively, compared to periodontitis-free individuals. Moreover, this risk escalates with increasing disease severity, and the magnitude of this association is modulated by factors such as disease severity, comorbidities, and oral hygiene status (38, 39).

### 2.5 Potential pathophysiological mechanisms

Beyond epidemiological correlations, the pathophysiological crosstalk between periodontitis and CVD primarily encompasses two pathways: direct vascular colonization by periodontal pathogens and indirect systemic perturbations triggered by periodontitis-driven inflammatory cascades. Specifically, periodontal pathogens exploit compromised gingival epithelial barriers to access to the systemic circulation, subsequently colonizing vascular walls. Their virulence determinants, such as lipopolysaccharide (LPS) and gingipains, disrupt complement components and their receptors, impair neutrophil bactericidal activity, and facilitate macrophage uptake of oxidized low-density lipoprotein (ox-LDL), thereby promoting foam cell formation and accelerating expansion of the atherosclerotic plaque lipid core. Additionally, molecular mimicry may trigger autoimmune responses targeting vascular tissues, such as the endothelium, further exacerbating vascular injury (19).

Moreover, chronic periodontal lesions release pro-inflammatory mediators that directly damage endothelial cells, inhibit endothelial nitric oxide synthase (eNOS) activity, reduce nitric oxide (NO) production, and elevate reactive oxygen species (ROS) and adhesion molecules such as vascular cell adhesion molecule-1 (VACM-1) and intercellular adhesion molecule-1 (ICAM-1). These effects promote monocyte adhesion and foam cell formation, accelerating AS progression. Additionally, they compromise vasodilation, increase vascular permeability, facilitate early atherosclerotic lesion development, and provide a pathological basis for acute myocardial infarction (AMI) (33). Periodontal pathogens and inflammatory mediators such as CRP and fibrinogen, can activate the coagulation system, elevate blood viscosity, and promote thrombosis. In individuals with existing atherosclerotic plaques, such processes may further increase stroke risk (38). However, the precise mechanisms underlying this association remain to be fully elucidated. Recent hypotheses propose that oral microbiota activate systemic humoral immunity including antibodies and complement system, which links localized periodontal inflammation to systemic vascular damage. This cascade, termed the “oral infection-immune dysregulation-vascular lesion” axis, constitutes a key biological basis for the association between periodontitis and CVD (40). Here, the periodontal lesion serves as a persistent inflammatory engine, driving systemic inflammation that primes the vascular endothelium and cultivates a pro-inflammatory microenvironment. This primed state endows the vasculature with exquisite sensitivity to subsequent inflammatory triggers, thereby accelerating the progression of stable AS toward acute clinical events, such as MI and stroke.

Overall, current research demonstrates that periodontitis represents a potential risk factor for CVD, contributing to the development of AS, CHD, MI, and stroke through both direct and indirect mechanisms. It is not only an independent risk factor for CVDs but also a pivotal component of the “oral-systemic health” axis. Clinically, acknowledging this association is imperative, and implementation of early screening, targeted periodontal interventions, and multidisciplinary collaboration is essential to mitigate the risk of cardiovascular events and optimize patient outcomes.

## 3 Diabetes mellitus

Beyond cardiovascular manifestations, the systemic impact of periodontitis extends to metabolic disorders, with DM representing one of its most well-characterized comorbidities. DM refers to a metabolic disorder of multifactorial etiology, defined by chronic hyperglycemia resulting from insulin secretion or action defects that disrupt carbohydrate, fat, and protein metabolism (41). The global prevalence of DM continues to rise, affecting 537 million adults in 2021 and projected to rise to 783 million by 2045, representing a substantial public health burden (42). As two prevalent chronic diseases in clinical practice, periodontitis and DM share a bidirectional interrelationship. DM elevates the risk of periodontitis, while periodontitis, in turn, impairs glycemic control and exacerbates diabetic complications (43). Accumulating evidence further substantiates this bidirectional association, underscoring their intertwined pathophysiology.

### 3.1 DM as a potential risk for periodontitis

Individuals with diabetes face 2- to 4-fold elevated risk of periodontitis relative to normoglycemic counterparts, with more severe clinical manifestations observed in this cohort. Glycemic level is a key determinant of this association (6, 43–45). Notably, type 2 DM shows robust correlation with periodontitis, particularly in middle-aged populations, while type 1 DM predisposes children and adolescents to accelerated periodontal destruction—with disease duration and suboptimal glycemic control further amplifying risk (41, 43, 46). Consequently, DM is recognized as a risk factor for periodontal disease progression, and glycated hemoglobin (HbA1c) levels are recommended for stratifying periodontitis severity (1, 47). Women with gestational DM (GDM) develop more severe periodontitis than those without GDM. A potential “inflammatory vicious cycle” exists between periodontitis and GDM, whereby hyperglycemia-induced oxidative stress and elevated placental extracellular vesicles and hormones exacerbate periodontal destruction and inflammation. Conversely, chronic periodontal inflammation amplifies systemic inflammatory cascades and immune dysregulation, aggravating insulin resistance and hyperglycemia, thereby promoting GDM and contributing to a bidirectional pathogenic cycle (43, 48). Animal studies further substantiate that insulin therapy can significantly attenuate inflammatory cell infiltration and cytokine gene expression in periodontal tissues of diabetic rats, thereby decreasing alveolar bone loss (49).

In DM, advanced glycation end-products (AGEs) accumulate in periodontal tissues. Interaction between AGEs and their receptors activates local immune and inflammatory responses. Upregulated inflammatory cytokines such as interleukin-1β (IL-1β), -6 (IL-6) and tumor necrosis factor-α (TNF-α), and exacerbate oxidative stress, disrupt the RANKL/OPG pathway, and promote alveolar bone resorption and periodontitis (41, 45, 50). Additionally, DM may enhance periodontal susceptibility by increasing the pathogenicity of periodontal microbiota and may be associated with increased IL-17 production (51, 52). Host susceptibility to periodontitis is influenced by periodontal microbiota, genetic factors, and lifestyle. Recent research suggests that DM can affect periodontal disease through epigenetic modifications (53). Hyperglycemia induced by DM causes epigenetic changes in 1,163 genes in gingival tissues, along with histological alterations, potentially increasing periodontal susceptibility in diabetic patients (54).

### 3.2 Impact of periodontitis on DM

Compared to individuals with healthy periodontal tissues, patients with periodontitis exhibit higher prevalence of elevated HbA1c, fasting blood glucose, and prediabetes. Furthermore, these patients face a 6-fold higher risk of poor glycemic control and diabetic complications (41, 50, 55). Evidence demonstrates that improving oral hygiene reduces the risk of incident type 2 DM (56). Non-surgical periodontal therapy (NSPT) lowers HbA1c levels in diabetic patients, with greater reductions observed in those with higher baseline HbA1c (57–59). Notably, treating periodontitis in T2DM patients also significantly mitigates systemic inflammatory marker levels, such as high-sensitivity CRP and IL-6, thereby alleviating chronic systemic inflammation (50, 60). However, while periodontal therapy shows promise in improving glycemic control among diabetic patients, the quality of current evidence remains limited, highlighting the need for further randomized controlled trials to validate these observations (61, 62).

The mechanisms by which periodontitis influences DM primarily involve the dissemination of periodontal pathogens and their products into systemic circulation. These agents can impair insulin signaling, increase insulin resistance, and ultimately lead to elevated HbA1c levels and diabetic complications (41, 45). This mechanism includes the inhibition of hepatic glycogen synthesis, the enhancement of hepatic gluconeogenesis, excessive production of branched-chain amino acids (BCAAs) that block insulin receptor substrates, the generation of dipeptidyl peptidase-4 (DPP4) which accelerates degradation of glucagon-like peptide-1 (GLP-1), thereby reducing GLP-1-mediated insulin secretion, and the induction of β-cell dedifferentiation, which alters insulin production. Systemic inflammatory burden induced by periodontitis can further promote insulin resistance in the liver and adipose tissue. Periodontal pathogens may also disrupt gut microbiota homeostasis, contributing to endotoxemia and alterations in blood metabolomics (52, 63).

The bidirectional crosstalk vividly exemplifies the systemic ramifications of the periodontal inflammatory engine. DM, via sustained hyperglycemia and AGE accumulation, reinforces the local persistence and pathogenicity of this periodontal engine. Conversely, the systemic dissemination of pro-inflammatory mediators from this engine exacerbates insulin resistance, a pivotal component of the systemic pro-inflammatory environment, thereby impairing glycemic control. This reciprocal amplification underscores the vicious cycle that lies at the core of this comorbidity. Although periodontitis and DM exert mutual influence, whether a causal relationship exists has yet to be definitively established via additional cohort studies. Given their strong bidirectional association, a multidisciplinary approach to managing patients with both conditions is therefore recommended to optimize disease control and patient outcomes.

## 4 Respiratory diseases

Respiratory diseases, both acute and chronic, rank among the leading causes of morbidity and mortality worldwide, encompassing conditions such as pneumonia, influenza, chronic obstructive pulmonary disease (COPD), obstructive sleep apnea (OSA), asthma, and lung cancer (64). The direct anatomical connection between the oral cavity and lungs provides a pathway for oral pathogens to translocate to the lungs, implicating a potential mechanistic link between periodontitis and respiratory diseases (65). Furthermore, diverse systematic reviews have consistently validated significant associations between periodontitis and pneumonia (65–68).

### 4.1 Coronavirus disease 2019 (COVID-19)

COVID-19, a highly transmissible respiratory disease caused by severe acute respiratory syndrome coronavirus 2 (SARS-CoV-2), utilizes angiotensin-converting enzyme 2 (ACE2) as its primary receptor—a molecule expressed in gingival epithelial cells, implicating a potential link between COVID-19 and periodontitis (69). Case-control study has identified a significant correlation between periodontitis and SARS-CoV-2 infection. Poor periodontal and oral hygiene may exacerbate COVID-19, with affected patients exhibiting more gingival bleeding and plaque accumulation (70). One case-control study found that the presence of periodontitis increases COVID-19 severity by approximately 3.7-fold (71). Meta-analysis of epidemiological studies further substantiates this association. Periodontitis may accelerate damage to the lower airway through direct inhalation of pathogens or indirectly exacerbate cytokine storm via chronic systemic inflammatory cascades, with SARS-CoV-2 possibly spreading through ulcerated gingival epithelium, leading to pulmonary vascular lesions (72).

### 4.2 Chronic obstructive pulmonary disease (COPD)

Like periodontitis, COPD is a chronic inflammatory disorder, and the two conditions share key risk factors such as smoking and aging. Clinical investigations reveal bidirectional interactions between them: COPD elevates periodontitis risk, with comorbid patients displaying exacerbated periodontal deterioration. Conversely, periodontitis is a potential risk factor for COPD, with its severity correlated with COPD mortality rates (73). Epidemiological evidence further demonstrates a positive correlation between periodontitis and COPD such that poorer periodontal status is associated with more severe COPD symptoms. Patients with severe periodontitis face a 3.5-fold higher risk of COPD, and periodontal treatment can enhance pulmonary function among COPD patients (74, 75). A MR analysis leveraging genetic prediction has revealed a potential causal relationship between periodontitis and COPD, indicating that periodontitis is an important risk factor for COPD. However, reverse MR analysis revealed no significant genetic causal association from COPD to periodontitis (76). Mechanistically, periodontitis may drive COPD progression through microbial interactions, immune imbalance, and systemic inflammatory responses. Periodontal pathogens can directly translocate to the lungs, where they damage the respiratory epithelium, induce pulmonary inflammation, and facilitate respiratory infections. Furthermore, periodontitis disrupts immune homeostasis and exacerbates COPD by inducing neutrophil-macrophage imbalance, dysregulating inflammatory cytokines, and perturbing circulatory immune-inflammatory responses (74).

### 4.3 Obstructive sleep apnea

OSA is a common sleep-related breathing disorder characterized by recurrent airway collapse during sleep, leading to intermittent hypoxia and systemic oxidative stress, which induce chronic inflammation and affect nearly one billion adults worldwide (77).OSA and periodontitis share multiple common risk factors, including age, smoking, alcohol consumption, DM, and obesity, indicating a potential association between the two conditions (78). Big data analysis suggests a possible positive correlation between periodontitis and OSA, with chronic inflammation in periodontal tissues potentially serving as a mediating factor in OSA-related inflammation (79). Conversely, multiple studies have demonstrated an increased prevalence and severity of periodontitis in patients with OSA, suggesting that OSA may act as a potential predictor of periodontitis (80–82). However, some studies found no significant association between OSA and periodontitis (83). Bidirectional MR analysis supports a causal effect of OSA on periodontitis, but there is no evidence indicating that periodontitis causes OSA (84). Given the high heterogeneity among studies and the relatively low quality of evidence, further research is needed to elucidate the association and causal relationship between these two conditions.

### 4.4 Asthma

Asthma, a chronic respiratory disease characterized by reversible airway obstruction and primarily driven by allergic or hypersensitivity reactions, affects over 300 million individuals worldwide, with its incidence rising (85, 86). Findings regarding the association between asthma and periodontitis remain conflicting. Some studies indicate a negative association, suggesting that asthma patients have a lower risk of severe periodontitis. Proposed mechanisms include healthier lifestyles and a dominant Th2 immune response that suppresses destructive osteoclastogenesis (87, 88). Summary-level data from genome-wide association studies (GWAS) using two-sample MR further support asthma as a potential protective factor against periodontitis, with reverse MR analysis yielding no evidence of a causal effect from periodontitis to asthma (89). Conversely, several case-control studies substantiate a positive crosstalk between periodontitis and asthma, with periodontitis patients exhibit a 3-fold elevated risk of severe asthma and the prevalence of periodontitis is elevated among patients with severe asthma compared to non-asthmatic individuals (90–92). Given these inconsistent findings, further research is warranted to clarify the complex relationship between asthma and periodontitis.

### 4.5 Lung cancer

Epidemiological studies indicates that chronic periodontitis is associated with an increased risk of lung cancer. Patients with chronic periodontitis exhibit a 2.3-fold higher incidence of lung cancer compared to individuals without the condition, with this risk elevation being particularly pronounced in middle-aged, non-smoking women (93, 94). Microbial and inflammatory milieus associated with periodontitis may contribute to lung carcinogenesis. These observations underscore a close link between oral and systemic health. Nevertheless, current evidence remains limited, and large-scale, well-controlled studies with adequate confounder adjustment are needed to substantiate this association more definitively.

### 4.6 Mechanisms of interaction

Oral microbiota plays a pivotal role in the interplay between periodontitis and respiratory diseases. Key pathogens such as *Actinomyces*, *P. gingivalis*, and *P. intermedia* are implicated in lung abscesses and pneumonia (66). The mechanisms underlying the influence of periodontal microbiota on the respiratory system include bacterial dissemination and colonization, alteration of mucosal environments, impaired pathogen clearance, and airway inflammatory responses (8, 95). First, periodontal pathogens may directly enter the respiratory tract via inhalation or saliva transmission, leading to infections, increased mucus secretion, and airway obstruction, particularly in immunocompromised individuals. Second, proteolytic enzymes secreted by bacteria degrade the oropharyngeal mucosal structure, exposing cellular receptors and facilitating pathogen adhesion. Third, salivary hydrolases such as sialidases, damage mucoproteins and disrupt biofilms, impairing pathogen clearance. Fourth, pro-inflammatory cytokines uch as IL-1β and TNF-α, released from periodontal tissues diffuse into the respiratory tract via saliva, where they stimulate epithelial cells, recruit inflammatory cells, induce epithelial damage, and heighten the risk of pathogen colonization (8, 66, 96). Conversely, respiratory diseases can exacerbate pathogen colonization by disrupting oral microbiota homeostasis and impairing immune function, thereby establishing a vicious cycle that aggravates respiratory pathology (95). Consistent with the pro-inflammatory engine framework, the periodontal engine operates through the physical aspiration of pathogenic microorganisms and the systemic dissemination of pro-inflammatory mediators that prime the pulmonary mucosal immune microenvironment. This primed environment not only heightens susceptibility to respiratory infection but also amplifies existing chronic inflammation in conditions like COPD and OSA. Importantly, respiratory diseases themselves can alter the oral microbiota and immune function, feeding back to fuel the local persistence of the periodontal engine, thereby reinforcing a bidirectional crosstalk between the oral cavity and the lungs.

To date, the evidence base linking periodontitis to respiratory diseases rests predominantly on observational data. Well-powered interventional trials are urgently needed to determine whether enhancing oral health can improve clinical outcomes in patients with respiratory diseases.

## 5 Preterm birth

Preterm defined as delivery before 37 weeks of gestation, remains a leading cause of perinatal death and disability in developed countries and a primary contributor to low birth weight (97, 98). Numerous risk factors for preterm birth have been identified, encompassing non-gynecological reasons such as maternal lifestyle habits and other systemic diseases, gynecological reasons like uterine malformations, uterine adhesions, and intrauterine infections during pregnancy, as well as obstetric factors (99). Periodontal disease has long been recognized as a risk factor for adverse pregnancy outcomes, with a significant association between maternal periodontitis and preterm birth (100, 101). Systematic review indicate that pregnant women with periodontitis face a 2-fold increased susceptibility of preterm birth compared to those without, though diagnostic criteria for maternal periodontitis remain inconsistent (102). A case-control study further validated a statistically significant association revealing that women with periodontitis had 6-fold higher risk of preterm birth (99). Additionally, studies have validated that women with preterm birth exhibit more severe periodontal attachment loss and probing depths than those with term birth, along with distinct compositional differences in supragingival and subgingival dental microbiomes between the two groups (103). However, other studies have reported no significant interplay between periodontitis and preterm birth after adjusting for confounding factors, suggesting that periodontitis may not act as an independent risk factor. These findings highlight the importance of maintaining periodontal health during pregnancy (104).

Bacteria and endotoxins from periodontitis can translocate hematogenously to the placenta, directly damaging placental tissue and entering the amniotic fluid to induce chorioamnionitis, thereby increasing the risk of preterm birth (105). Inflammatory mediators from periodontal lesions may also spread systemically, triggering immune-inflammatory responses in placental tissues. The subsequent release of pro-inflammatory cytokines into the amniotic fluid can further precipitate preterm birth (106). Alterations in microbial composition and specific pathogenic species may contribute to preterm birth, though the exact roles of these microbes, their interactions, and the impact of their metabolic products warrant further investigation (103). Moreover, spontaneous preterm birth and periodontitis share a genetic susceptibility to inflammatory phenotypes. Specific genetic variants in inflammation-related genes such as Toll-like receptor 1 (TLR1) rs5743618, may concurrently elevate the risk of both conditions (107). Collectively, these findings underscore a close link between periodontitis and preterm birth. Given its preventable and treatable nature, periodontitis represents an appealing modifiable target in obstetric care. Incorporating periodontal screening into routine prenatal visit may offer a low-cost, high-impact strategy to reduce the burden of preterm birth.

Viewed through our integrative framework, the pro-inflammatory milieu of periodontitis during pregnancy extends to the placental-fetal unit, a highly sensitive biological system, triggering preterm labor in hosts with inherent genetic or environmental susceptibility. This conceptualization reframes maternal periodontitis not as a direct cause of preterm birth, but as a modifiable amplifier of pre-existing inflammatory risk—a distinction that carries profound implications for prenatal screening strategies and clinical interventions.

## 6 Alzheimer’s disease

Alzheimer’s disease (AD) is a progressive neurodegenerative disorder marked by memory impairment, cognitive decline, and neuroinflammation, representing the most common form of dementia with a continuously rising global prevalence (108). Substantial evidence indicates a bidirectional association between periodontitis and cognitive decline (109). Patients with dementia exhibit a significantly higher risk of periodontitis, and those with early-onset dementia face an elevated future risk of developing periodontitis (110). Specific periodontal pathogens including *A. actinomycetemcomitans* and *P. gingivalis*, have been detected in the brains of AD patients, suggesting a potential mechanistic link between periodontitis and AD (111, 112). Patients with periodontitis have approximately a 1.7-fold increased susceptibility of developing AD. Elevated serum TNF-α levels and increased antibodies against periodontal pathogens such as *P. gingivalis, A. actinomycetemcomitans, T. forsythia, F. nucleatum, and P. intermediain* in AD patients further support this association (113). Notably, antibacterial or anti-inflammatory therapies targeting *P. gingivalis* can slow AD progression, indirectly corroborating the relationship (114). Prospective cohort studies have demonstrated that periodontitis may increase AD severity and accelerate disease progression. With AD advancement, both periodontitis severity and occlusal function deteriorate, underscoring a bidirectional dynamic (115). Collectively, these studies suggest that periodontitis is a modifiable risk factor for AD development. While periodontitis may influence AD progression, patients with AD have a heightened risk of periodontitis due to impaired self-care abilities, which compromises oral hygiene maintenance.

Current research indicates that *P. gingivalis* exerts a pivotal pathogenic role in the influence of periodontitis on AD, with neuroinflammation functioning as a critical mechanistic link between the two conditions (116). *P. gingivalis* and and its virulence factors such as LPS, can induce bacteremia and traverse the blood-brain barrier (BBB) to access the brain. LPS binds to microglia via Toll-like receptors 2 and 4 (TLR2/4), inducing the release of pro-inflammatory cytokine IL-1β, which activates neurons, upregulates the activity and expression of β-site amyloid precursor protein-cleaving enzyme 1 (BACE1), and exacerbates Aβ deposition (117). Subsequently, activation of β- and γ-secretases promotes the secretion of extracellular amyloid-beta peptides (AβP), which form oligomers and protofibrils that aggregate into amyloid plaques. These plaques are recognized by the triggering receptor expressed on myeloid cells 2 (TREM2) on microglial membranes, initiating an inflammatory cascade that further upregulates AβP production. LPS can also activates glycogen synthase kinase 3 (GSK-3), inducing hyperphosphorylation of Tau protein and the formation of neurofibrillary tangles (NFTs). Collectively, these processes drive neuronal damage, degeneration, and death, thereby propelling AD progression (118, 119). Additionally, the low-grade systemic inflammatory milieu associated with aging and periodontitis enhances BBB permeability, facilitating *P. gingivalis* entry into the brain. This process further compromises BBB integrity, permitting periodontal pathogens and virulence factors to penetrate the central nervous system and establish a vicious cycle (120, 121).

The periodontitis-induced systemic inflammatory response can also induce neurodegenerative alterations. Periodontal pathogens trigger excessive secretion of pro-inflammatory cytokines including TNF-α, IL-1β, and CRP, thereby establishing a chronic systemic inflammatory state. These peripheral pro-inflammatory factors can cross the BBB via systemic circulation to enter the central nervous system. Within the central nervous system, inflammatory signals are relayed to the brain through the leptomeninges, activating microglia to secrete IL-1β and upregulate β-site amyloid precursor protein-cleaving enzyme 1 (BACE1) activity, ultimately leading to neuronal dysfunction. Furthermore, peripheral pro-inflammatory factors can additionally elevate the levels of pro-inflammatory mediators in brain tissue via stimulation of trigeminal nerve fibers, promoting Aβ deposition and Tau hyperphosphorylation, inducing neuronal degeneration, and ultimately driving cognitive decline (116, 122–124).

In summary, the correlation between severe periodontitis and AD is well-established, with shared pathological pathways involving periodontal pathogens and systemic inflammatory cascades. Age-related immunosenescence and heightened BBB permeability define a specific neural susceptibility. In hosts with this inherent vulnerability, periodontal inflammation generates a systemic inflammatory milieu that facilitates neuroinflammation. This cascade reframes periodontitis not as a direct etiological agent of Alzheimer’s disease, but as a potent, modifiable driver that accelerates its pathological progression in susceptible individuals. Therefore, periodontal assessment should be integrated into routine evaluations to facilitate early detection and management of AD and potentially delay its progression.

## 7 Chronic kidney disease

Chronic kidney disease is defined by progressive structural and functional renal impairment, typically manifesting as a sustained decline in glomerular filtration rate (GFR). It affects approximately 9% of the global population, representing a significant public health burden (125, 126). Periodontitis and CKD share several common risk factors, including obesity, smoking, and advanced age, with accumulating evidence supporting an association between the two conditions (127, 128). Multiple studies demonstrate a bidirectional relationship that CKD patients exhibit a significantly higher prevalence of periodontitis, and periodontitis is associated with an increased risk of CKD development. Periodontal parameters show a negative correlation with renal function, with systemic oxidative stress identified as a key mediating factor (129–131). Notably, a large-scale prospective cohort study revealed an crosstalk between moderate-to-severe periodontitis and CKD-related mortality (132). A systematic review further substantiates that periodontal therapy (PT) may improve CKD status by mitigating systemic inflammatory burden (133). Analysis of National Health and Nutrition Examination Survey III (NHANES III) data established a significant positive correlation between periodontitis and CKD, with CKD patients facing a 2-fold elevated risk of periodontitis compared to individuals with normal renal function (134). Further large-scale intervention studies are required to clarify the causal relationship between periodontitis and CKD.

The mechanisms linking periodontitis to CKD primarily involve microbial, inflammatory, and oxidative stress pathways. Specific periodontal pathogens contribute to renal injury through distinct virulence factor-receptor interactions and downstream signaling cascades. *P. gingivalis* triggers inflammatory responses and renal damage via interactions of its fimbrial proteins, LPS, and gingipains with host receptors. Similarly, *A. actinomycetemcomitans* elicits destructive immune responses affecting renal tissues through interactions of peptidoglycan (PGN), outer membrane vesicles (OMVs), cytolethal distending toxin (CDT), and leukotoxin (LtxA) with host receptors. These interactions are mediated via pathogen-associated molecular patterns (PAMPs) and activation of nuclear factor-κB (NF-κB) and phosphoinositide 3-kinase (PI3K) signaling pathways. Another key pathogen, *F. nucleatum*, induces renal damage through interactions of its FadA adhesin and lipid A with host receptors, activating NF-κB, p38 mitogen-activated protein kinase (p38 MAPK), and mitogen-activated protein kinase (MAPK) signaling cascades (135, 136).

Inflammatory factors associated with periodontitis such as IL-1 and IL-6, induce the differentiation of CD4(+) T cells into T helper 17 (Th17) cells. The production of Th17-derived interleukin-17A (IL-17A) then stimulates renal tubular epithelial cells to produce monocyte chemoattractant protein-1 (MCP-1). Concurrently, IL-1 and TNF-α promote the expression of ICAM-1 by renal vascular endothelial cells and induce pentraxin 3 (PTX3) production by renal tubular epithelial cells, collectively facilitating renal thrombotic events. Additionally, periodontitis-induced elevations in plasma acute-phase proteins (APPs) and CRP, contribute to renal endothelial cell damage, exacerbating kidney disease (135). Periodontitis also triggers immune cells to generate reactive oxygen species (ROS), inducing lipid peroxidation and subsequent renal injury (130, 135).

In CKD patients, impaired renal function alters salivary composition and function, manifesting as elevated urea concentrations in saliva and changes in salivary pH, which subsequently alter the oral microbiota and increase the risk of colonization by periodontal pathogens (130, 137). This bidirectional crosstalk embodies the self-perpetuating nature of our model. The periodontal engine spreads microbial products and inflammatory mediators, creating a systemic pro-inflammatory milieu that makes kidneys highly prone to progressive injury. Conversely, CKD-induced uremia alters saliva and oral microbiome, fueling periodontitis. This cycle sees each disease drive the other’s inflammation, stressing the need for integrated renal and oral care.

The bidirectional relationship between periodontitis and CKD is currently recognized. However, the presence of multiple uncontrolled confounders in existing studies renders the causal relationship inconclusive, underscoring the need for large-scale intervention trials to elucidate causality.

## 8 Rheumatoid arthritis

Shared inflammatory pathways similarly link periodontitis to autoimmune joint disease, yet in rheumatoid arthritis, the oral cavity assumes an even more provocative role, not merely amplifying inflammation, but potentially initiating autoimmunity itself. Rheumatoid arthritis (RA) is a systemic autoimmune disorder driven by persistent synovitis, clinically presenting as symmetric joint swelling, pain, and progressive bone destruction (138). The relationship between periodontitis and RA is well-reported. epidemiological studies substantiate that RA patients exhibit a higher prevalence of periodontitis and more severe periodontal manifestations including increased probing depth, bleeding on probing (BOP), clinical attachment loss (CAL), and elevated inflammatory cytokine levels compared to healthy controls (139–141). Periodontitis is also considered a potential etiological trigger for RA. Following NSPT to reduce periodontal inflammation, RA patients showed significantly decreased clinical disease activity scores and CRP levels, suggesting that NSPT may serve as a potential adjunctive treatment for RA (142, 143). Furthermore, periodontitis and RA share common risk factors, including genetic predisposition such as HLA-DRB1-04, smoking, and infections with Epstein-Barr virus and cytomegalovirus (144). As chronic inflammatory disorders, both diseases feature pivotal roles for B lymphocytes and plasma cells in immune dysregulation. The clinical features also overlap, involving tissue destruction such as alveolar bone resorption and joint erosion, underscoring a potential mechanistic link between the two conditions.

Upon recognition of pathogen-associated molecular patterns (PAMPs) from periodontal pathogens by immune cells, these cells secrete pro-inflammatory cytokines, including IL-1β, TNF-α, and IL-6. Concurrently, bacterial phagocytosis and complement factor inhibition trigger apoptosis, inducing the release of damage-associated molecular patterns (DAMPs) Hematogenous spread of periodontal pathogens and inflammatory mediators then induces localized inflammation and chronic systemic inflammatory states. *P. gingivalis* can also induce the generation of novel citrullinated epitopes through its gingipains, molecular mimicry, and peptidyl-arginine deiminase (PPAD). This cascade subsequently induces the production of anti-citrullinated protein antibodies (ACPA) within periodontal tissues. ACPA enter systemic circulation, triggering autoimmune responses in synovial tissue and culminating in RA (145, 146). Meanwhile, the immune imbalance and chronic inflammation inherent to RA reciprocally aggravate periodontal tissue destruction and increase susceptibility to infection, establishing a bidirectional vicious cycle (147).

In summary, a significant correlation exists between periodontitis and RA, characterized by high comorbidity and shared pathogenic pathways. Periodontitis may act as a risk factor for RA onset and progression, while RA-associated immune dysregulation exacerbates periodontal breakdown. Periodontitis functions as a localized autoimmune ignition site, where *P. gingivalis*-driven citrullination generates ACPA that disseminate systemically to trigger synovial autoimmunity in genetically susceptible hosts, thereby establishing a self-amplifying oral-joint inflammatory axis. Thus, integrated management of both conditions is clinically imperative.

## 9 Helicobacter pylori (*H. pylori*) infection

As the anatomical entry point to the digestive system, the oral cavity allows direct translocation of microbes into the gastrointestinal tract via swallowed saliva, thereby modulating gut microbiota composition. Studies have substantiated that *H. pylori* can be detected in dental plaque biofilms, and Hp is a major pathogenic factor for gastritis, gastric ulcers, and gastric cancer (148). NHANES III data analysis revealed significantly associated with increased mortality risk from gastrointestinal cancers among individuals infected with *H. pylori (*149).A meta-analysis of observational studies further demonstrated a significantly elevated odds of *H. pylori* infection in patients with periodontal disease, with an odds ratio (OR) of 2.07 (150). Conversely, *H. pylori* infection increases the risk of periodontitis, and eradication *H. pylori* may reduce this risk (151). Although chronic periodontitis patients exhibit higher gastric *H. pylori* prevalence, studies have found no significant interplay between chronic periodontitis and *H. pylori* detection in subgingival plaque, gingival crevicular fluid, or gastric tissue (152, 153). Considerable controversy persists regarding the link between periodontitis and *H. pylori* infection. Current evidence is predominantly from observational studies with methodological limitations, and large-scale prospective cohort studies are warranted to establish a direct causal relationship. Periodontal disease serves as a persistent extra-gastric reservoir—a microbial engine that continuously seeds *H. pylori* into the gastrointestinal tract via swallowed saliva. Such chronic seeding can impair gastric mucosal defense, particularly in individuals with low gastric acid secretion or pre-existing epithelial damage. While cross-sectional evidence cannot confirm causality, our framework proposes testable hypotheses: effective periodontal therapy may reduce *H. pylori* recurrence after eradication, illustrating how this model directs translational and interventional research.

The above explorations into periodontitis and related systemic diseases are substantiated by multi-tiered credible evidence, as summarized in **Table 2**, which further validates the widespread and clinically relevant links between periodontitis and extraoral health impairments, strongly reinforcing our integrative framework centered on the periodontal inflammatory engine driving systemic comorbidities.

**Table 2:** Association between periodontitis and systemic diseases: evidence, credibility, and references.

Disease	Relationship with periodontitis	Evidence	Credibility	Refs.
CVD	Bidirectional association; periodontitis contributes to CVD via direct/indirect mechanisms.	Epidemiological studies, mechanistic studies, MR analysis, clinical interventions.	High: Multiple study types confirm association; periodontal treatment reduces CVD risk despite unproven direct causality for some subtypes.	(11, 12, 19, 22)
AS	Bidirectional association; periodontal pathogens promote plaque formation; No direct causality	Epidemiological studies, animal experiments, MR analysis, clinical interventions.	Moderate-high: Abundant epidemiological/mechanistic evidence; indirect effects via systemic inflammation supported.	(10, 14, 15, 19, 22, 23)
CHD	Moderate-severe periodontitis linked to prevalent/incident CHD; elevated serum antibodies against periodontal pathogens increase risk.	Epidemiological studies (cohort, case-control), serological studies.	Moderate-high: Sufficient epidemiological evidence; potential independent risk role but unproven direct causality.	(25, 28, 29, 31)
MI	Moderate-severe periodontitis correlates with acute MI risk (dose-dependent); stronger in females; exacerbates post-MI systemic inflammation.	Epidemiological studies, case-control studies.	Moderate-high: Clear epidemiological evidence with gender differences; reasonable inflammatory mechanisms.	(12, 32–34, 36)
Stroke	Moderate/severe periodontitis increases stroke risk by 1.71/2.55-fold; pathogens/inflammatory mediators activate coagulation.	Epidemiological studies, meta-analysis, mechanistic studies.	Moderate-high: Meta-analyses confirm dose-response relationship; pathological links supported.	(37–39)
DM	Bidirectional vicious cycle; DM increases periodontitis risk 2-4-fold; periodontitis impairs glycemic control and exacerbates complications.	Epidemiological studies, meta-analysis, animal experiments, clinical interventions.	High: Robust bidirectional evidence; periodontal treatment improves glycemic control and reduces inflammation.	(41, 43, 50, 55)
T2DM	Stronger bidirectional association; NSPT reduces HbA1c, especially in poor glycemic control.	Epidemiological studies, meta-analysis, clinical interventions.	High: Most well-evidenced DM subtype; consistent clinical intervention effects.	(46, 57, 58, 60)
GDM	Bidirectional inflammatory cycle; GDM exacerbates periodontitis; periodontitis aggravates insulin resistance/hyperglycemia.	Epidemiological studies, mechanistic studies, animal experiments.	Moderate: Bidirectional inflammatory links confirmed; fewer studies than T2DM.	(43, 48)
Respiratory diseases	Anatomical connection facilitates pathogen translocation; periodontitis increases pulmonary infection risk; bidirectional inflammation.	Epidemiological studies, meta-analysis, mechanistic studies.	Moderate-high: Clear anatomical/epidemiological basis; consistent meta-analytical evidence.	(65, 66)
COVID-19	Periodontitis increases severity by 3.7-fold; direct pathogen inhalation or cytokine storm exacerbation.	Case-control studies, meta-analysis, mechanistic studies.	Moderate: Retrospective studies confirm association; evidence quality limited by pandemic constraints.	(69–72)
COPD	Bidirectional association; periodontitis is a causal risk factor (MR-confirmed); periodontal treatment improves pulmonary function.	Epidemiological studies, MR analysis, clinical interventions.	High: Causality confirmed by MR; consistent epidemiological/clinical evidence.	(73–76)
OSA	Positive correlation trend; periodontal inflammation mediates OSA-related inflammation; high study heterogeneity.	Big data analysis, meta-analysis, MR analysis.	Low: Inconsistent findings; low-quality evidence for causal relationship.	(77, 79, 81, 84)
Asthma	Conflicting findings: asthma may be protective (Th2 immune suppression) or periodontitis increases severe asthma risk.	Case-control studies, observational epidemiological study, MR analysis.	Low: Contradictory evidence; MR supports asthma as a protective factor.	(87–89, 91, 92)
Lung cancer	Chronic periodontitis increases risk by 2.3-fold (more pronounced in middle-aged non-smoking women); microbial/inflammatory milieu contributes to carcinogenesis.	Epidemiological studies, meta-analysis, mechanistic studies.	Moderate: Target population evidence clear; limited overall study quantity.	(93, 94)
Preterm birth	Maternal periodontitis increases risk by 2-6-fold; pathogens/inflammatory mediators induce chorioamnionitis.	Epidemiological studies, meta-analysis, case-control studies.	Moderate: Most studies confirm association; inconsistent findings after confounder adjustment.	(97, 99, 100, 102, 105)
AD	Bidirectional association; periodontitis increases AD risk by 1.7-fold; Porphyromonas gingivalis induces neuroinflammation and Aβ/Tau pathology.	Epidemiological studies, case-control studies, mechanistic studies.	Moderate-high: Clear pathogen-mediated mechanisms; targeted therapy slows AD progression.	(110, 111, 113, 115, 116)
CKD	Bidirectional association; CKD patients have 2-fold higher periodontitis risk; moderate-severe periodontitis correlates with CKD-related mortality.	Epidemiological studies, meta-analysis, clinical interventions.	Moderate-high: Consistent bidirectional evidence; inflammation/oxidative stress pathways well-characterized.	(127, 128, 130, 132)
RA	High comorbidity; bidirectional vicious cycle; periodontitis is an etiological trigger; NSPT reduces RA disease activity.	Epidemiological studies, meta-analysis, clinical interventions.	High: Abundant comorbidity evidence; shared pathways and adjunctive benefits of periodontal treatment confirmed.	(139, 140, 142, 146, 147)
*H. pylori* infection	Bidirectional association; periodontal disease increases H. pylori risk (OR = 2.07); eradication reduces periodontitis risk; controversial subgingival/gastric tissue findings.	Epidemiological studies, meta-analysis, clinical interventions.	Moderate: Meta-analyses confirm association; methodological limitations in observational studies.	(148, 150–153)

## 10 Potential association mechanism between periodontitis and systemic diseases

**Figure 1:**
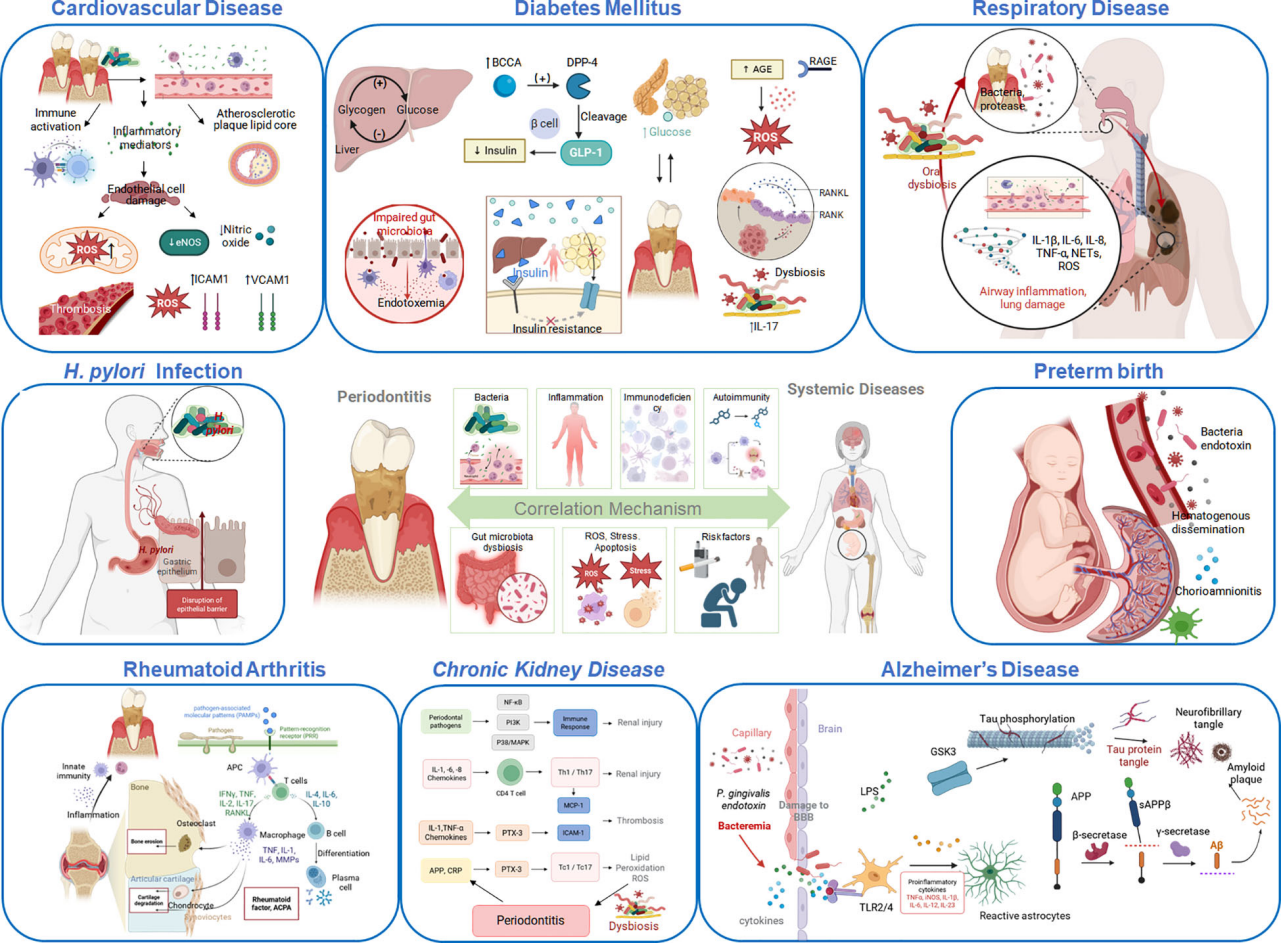
Association mechanism between periodontitis and systemic diseases.

Transcending its classification as a localized oral disorder, periodontitis serves as a key source of chronic low-grade inflammation and immune activation, intricately linked to the initiation, progression, and prognosis of diverse systemic diseases. Despite the pathological trajectories of these systemic conditions differ substantially, they converge with periodontitis through six core shared mechanisms (1): direct invasion and systemic dissemination of periodontal pathogens and their virulence factors (8, 154–156); (2) periodontitis-induced chronic systemic inflammation (8, 154, 155, 157) (3); immune dysfunction and aberrant activation (8, 154, 156, 157) (4); molecular mimicry and autoimmunity (158) (5); gut microbiota dysbiosis (158, 159) and (6) oxidative stress and cellular damage (158, 160, 161). Shared behavioral and risk factors including smoking, obesity, and psychological stress, promote the development of periodontitis while simultaneously exacerbating other systemic diseases (158, 162) (**Figure 1**).

The persistence of local inflammation ultimately leads to chronic systemic inflammation, which is a key mechanism connecting periodontitis with various systemic diseases (163). Sustained inflammatory state in periodontitis perpetuates systemic pathologies through maladaptive immune programs and self-sustaining feedback loops. Abnormal function of regulatory lymphocytes, maladaptive training immunity and clonal hematopoiesis of indeterminate potential (CHIP) are the core immune mechanisms, which form a bidirectional association between periodontitis and systemic inflammatory comorbidities such as RA, CAD, AD and type 2 DM (164, 165).

Regulatory lymphocytes, particularly Bregs and Tregs, act as immune gatekeepers in periodontal homeostasis. Bregs and Tregs maintain periodontal homeostasis by secreting anti-inflammatory cytokines (IL-10) to inhibit pro-inflammatory factors (IL-17, RANKL) and bone resorption. Their dysfunction disrupts immune homeostasis, failing to restrain excessive inflammation and further exacerbating periodontal destruction and systemic inflammatory burden (166). Maladaptive trained immunity is innate immune cells acquiring non-specific inflammatory memory via metabolic, transcriptional and epigenetic reprogramming of bone marrow HSPCs after microbial or inflammatory stimulation, with high reactivity upon re-stimulation. Periodontitis-related chronic inflammation triggers trained immunity via an inflammatory outbreak. Resultant trained neutrophils and monocytes aggravate local periodontal injury, infiltrate systemic tissues via the bloodstream, and contribute to comorbidities including AS and RA (165, 167).

Maladaptive trained immunity may serve as a mechanistic explanation underlying the correlation between periodontitis and chronic inflammatory diseases (168). CHIP, derived from aging-related somatic mutations in hematopoietic stem and progenitor cells (HSPCs), increases susceptibility to chronic inflammatory diseases including periodontitis, AS and RA (169, 170). Mutated HSPCs generate myeloid cells with enhanced inflammatory potential, which exacerbates periodontitis and promotes comorbidities. In turn, periodontal inflammation activates these pathways via hematogenous spread of pathogens and inflammatory mediators, establishing a bidirectional relationship (165). CHIP may provide a mechanistic basis for the involvement of innate immunity in linking periodontitis with other inflammatory comorbidities, yet this hypothesis remains to be fully validated.

The vicious cycle of dysbiosis, barrier dysfunction, and sustained immune activation related periodontitis that prevents return to homeostasis (171). Dysbiosis of the oral commensal microbiota directly undermines periodontal tissue barrier, creating a permissive microenvironment for microbial invasion. Pathogens and their metabolites exploit compromised epithelia junctions to infiltrate subepithelial tissues. Translocated pathogens engage pattern recognition receptors (PRRs) such as Toll-like receptors (TLRs) on resident dendritic cells or macrophages, triggering a cytokine storm characterized by IL-1β, TNF-α, and interferon-γ secretion. This pro-inflammatory milieu induces tissue destruction and systemic dissemination of microbial products, establishing a low-grade systemic inflammatory state (172–174). Circulating tissue breakdown products paradoxically fuel dysbiotic expansion by serving as nutrients for opportunistic pathogens, while simultaneously suppressing beneficial commensals via competitive exclusion. Moreover, sustained inflammatory signaling disrupts the host-microbe equilibrium. This tripartite interplay—dysbiosis-barrier dysfunction-immune activation—constitutes a self-perpetuating positive feedback loop, keeping periodontal tissues and the systemic circulation in a state of chronic inflammation for a long time, thereby inducing systemic diseases such as RA and CVD (4, 158, 171, 175, 176).

As a chronic oral inflammatory condition, periodontitis establishes a bidirectional association with multiple systemic chronic inflammatory diseases, including AS, RA, and DM, through core immune mechanisms. Targeting these immune mechanisms and key inflammatory mediators represents a promising strategy for the integrated management of periodontitis and its associated systemic comorbidities.

## 11 Conclusion

Periodontitis is far more than a localized oral condition, it is a silent, modifiable and clinically actionable driver of systemic inflammation. A growing body of evidence linking periodontitis to multiple systemic diseases underscores the urgent need for integrated oral-systemic health management in clinical settings. Future research must prioritize three key domains, mechanistic explorations, targeted interventions, and interdisciplinary collaboration, to elucidate causal relationships and guide the development of effective preventive strategies.
